# The patient as teacher: a four-phase blueprint for institutional change

**DOI:** 10.3389/frhs.2026.1920768

**Published:** 2026-08-10

**Authors:** Lucas Wollmann, Axel Wolf, Lara Cooke, Maria Santana

**Affiliations:** 1 Department of Community Health Sciences, University of Calgary, Calgary, Alberta, Canada; 2 Centre for Person-Centred Care (GPCC), Sahlgrenska Academy, University of Gothenburg, Gothenburg, Sweden; 3 Department of Clinical Neurosciences, University of Calgary, Calgary, Alberta, Canada

**Keywords:** health profession education, implementation science, institutional change, patient educator, patient involvement, patient-centered care, social accountability

## Abstract

**Introduction:**

Over recent decades, patient involvement in medical education has shifted from passive case demonstrations toward active partnerships in teaching, assessment, and curriculum design, a transition that must recognize patients and relatives as persons with experiential knowledge, not merely as educational instruments. This perspective article argues that accumulated evidence now justifies a deliberate shift from scattered, champion-driven initiatives to embedded institutional strategy.

**Evidence and gaps:**

The evidence supports meaningful benefits across stakeholders: learners develop stronger clinical reasoning, communication skills, and empathy; patient educators report empowerment and enhanced self-esteem; and institutions advance their social accountability mission. However, the field continues to struggle with inconsistent terminology, fragmented implementation, short-term satisfaction-based outcomes, underexplored ethical considerations, and weak institutional commitment.

**Proposed roadmap:**

To address these gaps, we propose a four-phase implementation roadmap. Phase 1 (Institutional Diagnosis) maps current practices against Towle et al.'s six-level taxonomy and establishes an institutional position statement. Phase 2 (Role Definition and Phased Goal-Setting) advocates stepwise progression, typically starting at Levels 3–4, where patients share experiences or act as trained teachers, advancing toward full curriculum governance partnership. Phase 3 (Implementation Planning and Capacity Building) addresses curriculum integration, resource allocation, faculty development, and community partnerships. Phase 4 (Outcomes Monitoring and Evaluation) calls for multilevel, longitudinal evaluation frameworks, including systematic assessment of patient educators’ experiences.

**Discussion:**

We present this roadmap as an evidence-informed conceptual synthesis whose implementation depends on continued institutional commitment. Cross-cutting principles include incrementalism, sustained institutional commitment, patient co-production, representative recruitment, and rigorous longitudinal research.

## 1 Introduction

The idea that patients can actively contribute to medical education is not new. William Osler's oft-cited dictum that the best teaching is that taught by the patient himself (1) captures an intuition that has guided clinical education for over a century. Yet for most of that history, patients in medical educational settings were treated as passive, interesting cases to be observed rather than voices to be heard.

This began to change over recent decades, as educational initiatives increasingly incorporated patients into active teaching roles, particularly in clinical skills. The scope of patient involvement in health professional education has since expanded considerably (2–4), spanning a spectrum of roles from sharing lived experience to partnership in curriculum design, assessment, governance, and evaluation. We use patient involvement as an umbrella term, while recognizing that Patient and Public Involvement (PPI), in its fuller sense, requires influence over decisions and not merely presence in educational activities. A landmark review by Towle and colleagues synthesized approximately 270 papers spanning nine countries and proposed a taxonomy ranging from paper-based patient scenarios (Level 1) to full institutional partnership in curriculum development and governance (Level 6) (3). More recently, Dijk and colleagues systematically reviewed 49 empirical studies and documented an increasingly diverse range of patient roles, settings, and learning objectives in undergraduate medical education (5).

The rationale for this shift is well-grounded. Driving forces include the broader movement toward patient-centred care and shared decision-making, and growing recognition that patients, as experts in their own illness experiences, bring a form of knowledge that faculty and simulated patients cannot replicate (6, 7).

This rationale is reinforced by evidence on how such knowledge changes learners. A realist review by Bansal and colleagues demonstrated that meaningful engagement with real patient narratives, when combined with person-centred theory and structured reflection, is associated with perspective transformation toward person-centredness in learners, an outcome that communication skills training alone consistently fails to achieve (8).

Yet despite this evidence, patient involvement in medical education remains largely opportunistic, underfunded, and insufficiently evaluated. Most programs are one-off events initiated by individual champions rather than embedded in institutional strategy (3–5, 9, 10). Terminology remains inconsistent, complicating synthesis (11), and the long-term effects on healthcare practice and patient outcomes remain poorly understood (3, 9). While modern medicine claims to be patient-centred, institutional and educational structures remain overwhelmingly provider-centric.

This perspective article makes the case that the evidence now supports moving from scattered initiatives to systematic institutional implementation, and proposes a four-phase implementation roadmap to guide institutions through this transition in a deliberate, stepwise, evidence-informed manner. In doing so, we build on, but differ from, existing work: whereas Towle et al.'s taxonomy classifies degrees of involvement (3), evidence syntheses such as Gordon et al.'s BEME review appraise what has been done (4), and practical guides such as Eijkelboom et al.'s twelve tips operate largely at the programme level (10), our contribution reframes the taxonomy as a developmental trajectory and embeds it in an institution-level change-management sequence, from diagnosis to evaluation.

## 2 The evidence base: benefits, breadth, and remaining gaps

### 2.1 What the evidence supports

The case for patient involvement rests on converging evidence across stakeholder perspectives.

From the learner's perspective, studies consistently report high satisfaction with patient-led teaching. Real patient contact supports the development of clinical reasoning, communication skills, empathy, and contextual understanding (1); students’ attitudes toward chronic illness, disability, mental health, and aging improve meaningfully after patient contact. Real patients are perceived as more authentic than simulated counterparts and rated as more useful for practising clinical and communication skills (1, 3, 5). Trained patient educators can achieve learning outcomes equivalent to faculty in musculoskeletal examination teaching, while adding immediate, experiential feedback (3, 5). Longitudinal programs, in which students follow a patient over time, are particularly associated with a holistic, person-centred perspective (8).

From the patient's perspective, involvement is experienced as meaningful and empowering. Patients report raised self-esteem, a more coherent illness narrative, and deeper insight into the healthcare system (3, 12); some describe practical benefits, such as patients with back pain who reported improved self-management after teaching students (5). Retention and satisfaction are most robust in programs that involve patients in planning, formally acknowledge their contributions, and provide ongoing training and support (3).

From the institutional and societal perspective, patient involvement is increasingly framed as a component of social accountability, the obligation of medical schools to direct their activities toward the priority health needs of the communities they serve (13, 14). The World Health Organization framework articulated by Boelen and Heck holds that relevance, quality, cost-effectiveness, and equity must be pursued across education, research, and service (15); active patient involvement advances each of these values.

These benefits, however, are unevenly evidenced. The strongest support concerns learner-level outcomes and patients’ own accounts of their experience; evidence for sustained behavioural change in practice is sparse, and effects on patient health outcomes have not been rigorously demonstrated (3–5, 9). The claim that involvement advances an institution's social-accountability mission remains largely conceptual, resting on alignment with recognised values rather than measured institutional-level impact (13–15).

### 2.2 Persistent gaps and challenges

Despite this encouraging picture, substantial challenges remain. The literature is fragmented: inconsistent terminology makes synthesis difficult. A narrative review by Burnier and colleagues identified at least six distinct terms (patient educator, instructor, teacher, partner patient, mentor, and volunteer patient) in use, often with conflicting definitions (11), limiting transferability and impeding common standards.

Methodological limitations are pervasive. Most studies rely on self-reported learner satisfaction measured immediately after the encounter; few assess behavioural change in practice, none rigorously examine long-term patient outcomes, randomized trials are rare, and most studies lack control groups (3–5, 9).

Ethical considerations are inadequately addressed in most published studies. Beyond consent and confidentiality, sharing an illness narrative can carry a genuine emotional burden: patients have reported feeling exposed, objectified, or exploited, and longitudinal roles can become distressing when a contributor's own health deteriorates (16). Emotional safety is therefore a core design requirement, not a peripheral courtesy. Adjacent fields offer guidance: work on trauma-informed and intersectional engagement argues that partnerships should rest on trust, transparency, and critical reflexivity so that recounting difficult experiences does not re-traumatise contributors (17). A fuller ethical framework would translate these principles into fair compensation, structured preparation and debriefing, emotional safety, and an explicit right to withdraw without explanation, while guarding against testimonial extraction, the use of narratives for educational effect without reciprocal benefit, support, or genuine influence. Experiential knowledge should be recognised as legitimate and irreplaceable, not merely illustrative material (18).

Compensation warrants specific attention, because it signals whether an institution treats contributors as partners or unpaid resources. Guidance now exists to move beyond *ad hoc* arrangements: a recent scoping review catalogues policies and models for recognising patient partners through non-financial (authorship, formal acknowledgement) and financial (honoraria, hourly rates) means, while noting that compensation is still rarely reported or standardised (19). Financial sustainability is likewise under-examined; although patient-led teaching may be cost-effective relative to faculty-led equivalents, no study has conducted a rigorous cost-benefit analysis (3–5, 9).

Finally, who is invited to teach shapes what learners regard as a typical patient. Programmes often recruit contributors chosen for communication skills or clinical stability, with consequences: this can over-represent articulate, stable, and more advantaged individuals while screening out those whose conditions are complex, fluctuating, or stigmatised, precisely the populations whose perspectives most challenge learners and best reflect the social-accountability mission (13–15). Left unexamined, such selection reproduces the “usual suspects” pattern documented across patient and public involvement, concentrating participation within a narrow group while the least-heard voices remain absent (20). Countering it requires deliberate outreach to seldom-heard groups, partnership with community organisations, and trauma-informed support, so that participation does not depend on a contributor's pre-existing confidence or robust health (17).

Institutional commitment, too, remains the exception. Most initiatives are designed and sustained by individual faculty, leaving them vulnerable to discontinuation, and few institutions have embedded patient involvement in governance structures, strategic plans, or accreditation frameworks (3–5, 9).

## 3 A roadmap for implementation

**Figure 1:**
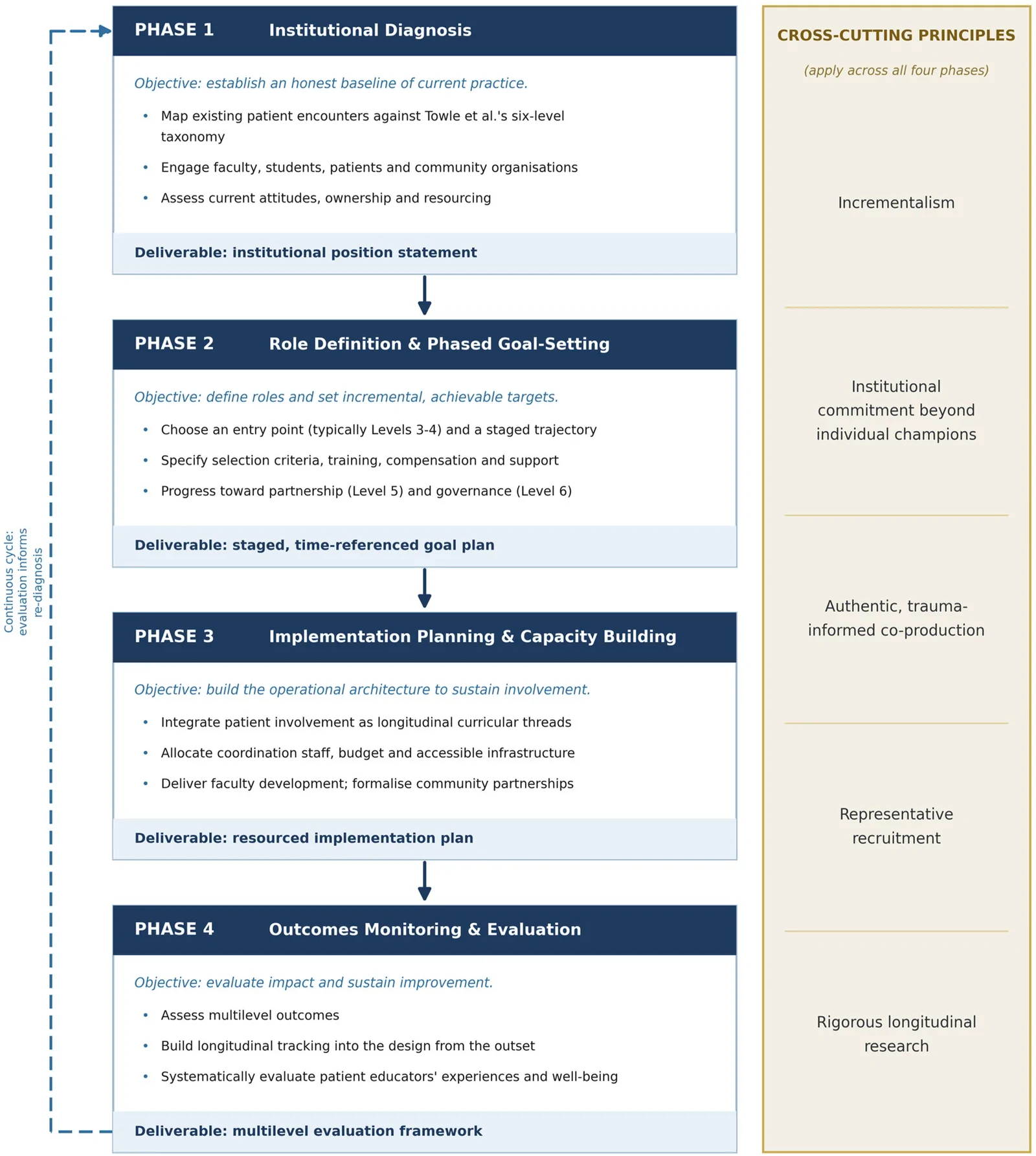
The four-phase roadmap for embedding patient involvement in health professional education. Each phase lists its central objective, representative activities, and principal deliverable; the cross-cutting principles apply throughout, and evaluation feeds back into diagnosis as a continuous cycle.

We propose that institutions approach patient involvement as a deliberate, phased transformation rather than a collection of *ad hoc* initiatives. The roadmap below consists of four sequential phases, each with specific objectives, activities, and deliverables. Figure 1 summarises the four phases, their objectives and deliverables, and the principles that run across them.

### 3.1 Phase 1: institutional diagnosis

Before designing any program, institutions must honestly assess where they stand. This diagnostic phase maps existing practices: which patient encounters already exist, whether patients play active or passive roles, who owns these initiatives and how they are resourced, and what attitudes prevail among faculty and leadership.

This assessment should engage multiple stakeholders, faculty, students, patients, and community organizations, and be guided by an established framework. Towle et al.'s six-level taxonomy provides a useful lens: institutions can map current activities against this spectrum and identify the gap between present practice and desired future state (3).

The phase should conclude with a clear institutional position statement articulating the rationale and commitment to patient involvement as a sustained educational priority.

### 3.2 Phase 2: role definition and phased goal setting

With a clear picture of the current state, institutions must define what they want involvement to achieve and what roles patients will play. We advocate a stepwise approach, beginning with simpler, lower-risk roles and progressing toward more complex partnerships as capacity, experience, and trust develop.

Towle et al.'s taxonomy (3) again provides a practical progression framework (Table 1). For institutions beginning this journey, Levels 3 and 4 are achievable, impactful starting points: inviting patients to share experiences within faculty-directed sessions, or training patient-teachers to teach and give feedback on clinical skills. These roles are well-supported by evidence, have established training models, and carry manageable ethical and logistical complexity (3, 21, 22).

**Table 1:** Towle et al. taxonomy of patient involvement in health professional education, with proposed implementation guidance.

Level	Description	Recommended starting point	Progression considerations
1	Paper-based or electronic case	Foundation (already in most curricula)	–
2	Standardized/volunteer patient in scripted clinical encounter	Common entry point	Ensure consent and adequate preparation
3	Patient shares experience within faculty-directed curriculum	Recommended first active role	Brief training, clear framing, faculty support
4	Patient-teacher involved in teaching and/or evaluating students	Core intermediate goal	Structured training, formal recognition, feedback mechanisms
5	Patient-teacher as equal partner in education, evaluation, and curriculum development	Advanced goal	Sustained infrastructure, compensation, governance representation
6	Patient involved at institutional level across educational continuum	Aspirational endpoint	Formal institutional policies, decision-making authority

Adapted from Towle et al. (3).

As capacity and confidence grow, progression to Level 5, patients as equal partners in education, evaluation, and curriculum development, becomes possible. This requires sustained infrastructure, dedicated patient liaison roles, formal governance representation, and adequate compensation and recognition. Level 6, involving patients in institutional decision-making across the educational continuum, is the aspirational endpoint for mature programs.

Role definition should also specify selection criteria, training requirements, compensation models, and mechanisms for ongoing support and feedback (3, 10). Training reduces patient anxiety, improves teaching quality, and signals institutional respect; even brief, well-structured orientation makes a meaningful difference (2, 3, 5).

### 3.3 Phase 3: implementation planning and capacity building

With roles defined and goals set, institutions can design the operational architecture to support involvement sustainably, across several interconnected activities.

Curriculum integration requires identifying where involvement will be embedded across the educational continuum, not as isolated events but as longitudinal threads through preclinical and clinical training. Bansal and colleagues’ realist review suggests person-centred learning is most transformative when integrated with explicit person-centred theory and structured sense-making in small groups (8). Institutions should resist adding involvement as a supplementary module; it should be a core curricular component aligned to programme-level outcomes.

Beyond where it sits in the curriculum, integration needs a conceptual anchor. A useful anchor is the triad in the Gothenburg Person-Centred Care (GPCC) framework: patient narratives engaged as knowledge sources revealing resources, capabilities, suffering, goals, and context; patients involved as partners in shaping objectives, methods, and evaluation; and educational agreements, roles, expectations, and outcomes formally documented and revised over time (18).

Infrastructure and resources must be addressed explicitly: dedicated coordination staff, budgets for training and compensation, accessible facilities, and systems for communication and feedback. Done well, involvement is resource-intensive; institutions that treat it as a cost-free add-on produce tokenistic programs that harm rather than advance patient-centred education (4, 5, 10).

Faculty development is often overlooked but essential. Attitudes vary widely, with some faculty concerned about patient vulnerability, the reliability of patient assessment, or dilution of their expertise (3, 5). Exposure to the evidence base and to effective patient-led teaching can shift these attitudes and build facilitation skills.

Community and organisational partnerships with advocacy groups, community organizations, and health networks support recruitment and retention, bring credibility, and increase diversity of representation (5, 10). Institutions should formalize these through memoranda of understanding or advisory committees rather than informal connections.

### 3.4 Phase 4: outcomes monitoring and evaluation

The fourth and most neglected phase involves designing a systematic evaluation framework from the outset. Without it, programs cannot demonstrate value, identify improvements, or contribute to the evidence base.

Outcome frameworks should span multiple levels, drawing on established models such as Kirkpatrick's hierarchy (reaction, learning, behaviour, results), Guskey's model of evaluating professional development (23), and national Competency-Based Medical Education (CBME) frameworks. At minimum, institutions should monitor learner satisfaction and perceived relevance, learning outcomes across competency domains, and patient experience and well-being; as programs mature, evaluation should extend to behavioural change in practice and, ultimately, patient care quality and health outcomes.

Longitudinal evaluation is especially important and especially rare. Since most studies assess outcomes immediately post-encounter, there is an urgent need for data on whether benefits persist across the continuum and into practice (3, 4). Institutions that build longitudinal tracking in from the start will make a distinctive contribution.

The experiences of patient educators deserve particular attention: their sense of contribution, psychological well-being, perception of institutional respect, and the adequacy of preparation, debriefing, and compensation, all both an ethical obligation and a predictor of sustainability (2, 3, 5). Instruments to capture these should be developed in partnership with patients.

## 4 Discussion

Active patient involvement in medical education has accumulated enough evidence to justify a transition from individual innovation to institutional strategy, with real benefits for students, patients, and the health systems that need practitioners capable of genuine partnership (2–5). What has been missing is an actionable framework for implementation; the roadmap proposed here provides a structured sequence adaptable to any institution's context, resources, and aspirations.

A number of principles cut across all four phases. First, incrementalism is a virtue: institutions that leap from passive encounters to full curricular partnership tend to meet resistance, logistical failure, and ethical risk, whereas building iteratively from well-supported, lower-complexity roles is more sustainable. Second, involvement must rest on institutional commitment rather than individual champions, requiring visible leadership endorsement, dedicated resources, and integration into accreditation and quality-assurance processes. Third, patients must be genuine partners from the earliest stages, not recruited to execute faculty-determined plans; as set out above, this entails fair compensation, preparation and debriefing, emotional safety, representative and trauma-informed recruitment, and a real right to withdraw. The distinction between tokenism and authentic co-production is ethically and educationally fundamental.

Patient involvement should also be understood as a governance and implementation strategy, not merely a pedagogical method. In concrete terms, the governance structures that sustain it include reserved and, ideally, voting seats for patients and community members on curriculum, assessment, and programme-evaluation committees; a standing patient (or patient-and-family) advisory council with a formal reporting line into curriculum governance rather than an informal consultative role; patient co-chairs for specific initiatives; a dedicated patient-partner liaison post; and explicit inclusion of involvement in strategic plans, quality-assurance cycles, and accreditation self-studies. Because accrediting bodies shape every programme, embedding expectations for patient involvement in their standards may be among the most powerful system-level levers available, though most agencies currently do little to require it (24). Engaging patients as genuine partners from the earliest stages prepares future professionals for health systems where patients and relatives are legitimate partners in care, improvement, research, and organisational development. Bergholtz and colleagues have shown how person-centred frameworks can reduce tokenism by embedding narrative, partnership, and structured documentation into institutional practice (18), offering actionable guidance for institutions moving beyond tokenism.

The field also needs investment in rigorous, longitudinal research. The dominance of short-term satisfaction surveys is a profound limitation; multi-institutional collaborations, qualitative research, longitudinal cohort designs, and good outcome instruments for patient educators are priorities that individual institutions cannot address alone.

A caveat must frame these recommendations. The roadmap is a conceptual synthesis: it organises existing evidence, frameworks, and reasoning into an actionable sequence, but it has not itself been implemented or evaluated, and the proposition that embedding involvement improves institutional or patient-level outcomes remains untested. Its phases and principles are best understood as hypotheses for institutions to adopt and study, not as claims backed by demonstrated institutional results, a reason to build evaluation into implementation from the outset, so that adoption itself generates the evidence the field now lacks.

## 5 Conclusion

Patient involvement in health professional education has reached a point where the binding constraint is no longer evidence or conceptual clarity but institutional will and design. The four-phase roadmap offered here, from diagnosis through phased role definition and capacity building to evaluation, provides one structured, adaptable path from scattered, champion-dependent activity toward embedded institutional practice, held together by incrementalism and authentic co-production, representative recruitment, durable governance, and rigorous longitudinal research. Although the evidence base is strongest in undergraduate medical education, the same principles extend across nursing, medicine, rehabilitation, social work, pharmacy, and other health and welfare professions. Treating patients’ perspectives and lived experiences as a foundational component of education, and testing that commitment empirically as it is enacted, is essential preparation for clinicians who can practise genuinely person-centred care. The evidence, frameworks, and rationale are in place; what remains is the institutional will to act, and the resolve to generate the evidence that will show whether, and how, this investment pays off.

## Data Availability

The original contributions presented in the study are included in the article/Supplementary Material, further inquiries can be directed to the corresponding author.
